# A sociology of anxiety: Western modern legacy and the Covid-19 outbreak

**DOI:** 10.1177/0268580921993325

**Published:** 2021-07

**Authors:** Paola Rebughini

**Affiliations:** Department of Social and Political Sciences, University of Milan, Italy

**Keywords:** Anxiety, Covid-19, emotions, Europe, expert knowledge, Anxiété, Covid-19, émotions, Europe, savoir des experts

## Abstract

The aim of this article is to investigate the role of anxiety as an emotional state frequently identified as the most characteristic of the history of modern Europe, and of the ‘Global North’. Starting from an assessment of the main supporters of this interpretation in social theory – such as the risk society approach of Ulrich Beck, and the role of expert knowledge in Anthony Giddens – the article discusses the current relevance and limitations of this well-established notion. The second part applies this discussion to the role of anxiety in the recent Covid-19 outbreak, and more specifically with regard to its relations with trust in scientific knowledge. Even though Covid-19 has been a global pandemic, this emergency can reveal some cultural and historical characteristics of European anxiety in the geo-cultural map of emotions.

## Résumé

L’objectif de cet article est d’étudier le rôle de l’anxiété en tant qu’état émotionnel fréquemment identifié comme le plus caractéristique de l’histoire de l’Europe moderne et du « Nord global ». En partant d’une évaluation des principaux tenants de cette interprétation dans la théorie sociale – comme la conception de la société du risque d’Ulrich Beck, et le rôle du savoir des experts chez Anthony Giddens – l’article examine la pertinence et les limites actuelles de cette interprétation bien établie. La deuxième partie applique cette analyse au rôle de l’anxiété dans l’épidémie récente de Covid-19, et notamment à ses relations avec la confiance dans les connaissances scientifiques. Même si le Covid-19 est une pandémie mondiale, l’urgence de la situation peut être révélatrice de certaines caractéristiques culturelles et historiques de l’anxiété européenne dans la carte géoculturelle des émotions.

## Resumen

El objetivo de este artículo es investigar el papel de la ansiedad como un estado emocional frecuentemente identificado como el más característico de la historia de la Europa moderna y del “Norte global”. A partir de una evaluación de los principales defensores de esta interpretación en la teoría social, como el enfoque de la sociedad del riesgo de Ulrich Beck y el papel del conocimiento experto en Anthony Giddens, el artículo analiza la relevancia actual y las limitaciones de esta interpretación establecida en la literatura. La segunda parte aplica esta discusión al papel de la ansiedad en el brote reciente de Covid-19 y, más específicamente, a su relación con la confianza en el conocimiento científico. Si bien el Covid-19 es una pandemia global, esta emergencia puede revelar algunas de las características culturales e históricas de la ansiedad europea en el mapa geocultural de las emociones.

## A geo-cultural contextualization of anxiety

With some notable exceptions (Beck, 1992; Giddens, 1990), social sciences have not paid specific attention to anxiety; other closer emotions such as anger or fear, but also love and compassion, have been more often investigated as mobilizing factors, in individual as well as collective action (Bericat, 2016; Goodwin et al., 2001; Scribano, 2020). Nonetheless, anxiety is a common emotional state, frequently present in everyday life, often responsible for physical discomfort, and correlated not only with contingent situations but also with cultural dispositions and orientations (Davidson et al., 2005; Harding and Pribram, 2002).

Anxiety is a slippery emotion. As well as being a buzzword, it is a common everyday experience, pervasive and sometimes unnoticed because of its low intensity, or its continuity, compared to other emotional upheavals. Anxiety is not necessarily a temporary state, or the emotional response to a situation. It is different from fear, as a specific and present threat: anxiety does not stem from a specific object and it is not something from which one can escape as in the case of a temporary danger. It can also be a permanent emotional condition, a background emotional state, typically aroused by moments of uncertainty, unpredictability, judgement, competition, necessity to take a decision. In many situations, anxiety is a typical response to stress, and in the psychological literature it is described as an amorphous ‘emotional disorder’, a lack of equilibrium and serenity subject to clinical diagnosis (Brosschot et al., 2016). Overall, anxiety is considered to be an emotional state of worry, apprehension, concern, vaguely associated with feelings of menace.

In Western culture, anxiety is also a typically future-oriented emotion (Melucci, 1996), and it is correlated to the feeling of trust since they both have a relation with the openness of the future typical of modernity. As Giddens noted (Giddens, 1991), future-oriented modernity fosters trust in expert systems, but also reflexivity, criticism and anxiety towards them. Modernity recognizes that the future is unknowable, while at the same time it is seen as an area of possibility, thus open to risk calculation, self-monitoring of global risks, and self-reflexivity towards expert knowledge, producing an anxiety related to the tendency to trust no one and become a self-expert by drawing on contradictory sources of information.

Indeed, anxiety, like all emotional states, has a cultural and not only subjective expression. Every individual is socialized to a certain way to express it, and anxiety can be allowed or repressed according to the cultural traditions and the rules of the context. For example, in Confucian-based culture anxiety is associated mainly to achievement and performance, more than to future orientations and risk calculation, and this has not any specific relations with modernity and modernization (Chen and Huttal, 1988; Chua, 2011). The cultural frame can not only influence the expression of emotions and the way in which they shape social relations; it can also foster some kinds of emotions rather than others (Davidson et al., 2005; Mesquita and Boiger, 2014). The cultural space where the individual lives cultivates specific emotional patterns and meanings given to them.

Hence, whilst anxiety as a temporary feeling of worry and apprehension can be found in any time and any place, in every culture and in every age, it has been considered to be a collective emotional state especially rooted in the history of Western modernity, and today the emotion on the global scale *par excellence*. Anxiety is the inseparable companion of uncertainty; and the focus on uncertainty has especially characterized the economic processes of globalization of recent decades. Anxiety is not only an individual emotional state related to everyday life situations; it is also the distinctive emotional feature of a globalization fostered mainly under the influence of Western ideas of uncertainty and risk (Beck, 2005), as well as of the necessary trust in expert systems (Giddens, 1990). In a sense, the typical relationship of Western modernity with anxiety can be considered a cultural attitude exported with a globalization shaped mainly by its first ‘Western impulse’ (Spivak, 1999).

Taking into account the geo-cultural variability of the expression of anxiety, the first part of this article examines the link between Western culture and anxiety. Anxiety is a relatively specific emotional and cultural phenomenon, and in Europe it is at the core of typical twentieth-century disciplines like psychoanalysis. Western societies are based on a historical culture of rationality, control, and teleological hope in never-ending progress. They have a low tolerance of uncertainty, and this has fostered a continuous search for a cause, a culprit, an enemy, to be removed so that a situation of control can be re-established (Giddens, 1990). Moreover, what is a matter of concern and a cause of anxiety in the Western world continues to set the agenda of global worries with systemic effects. Such sources of public anxiety are usually related to governance of the immediate future: managing immigration flows, forecasting economic upheavals, governing poverty, social exclusion and political unrest, monitoring technoscience research, estimating environmental catastrophes and climate change, to name but a few. Such anxieties are usually expressed and monitored in terms of risk, and the way in which they are expressed and managed can change according to the generation and category of people concerned, as well as to the local context and its characteristics (Beck, 2009).

The second part of this article will analyse the pandemic crisis of Covid-19 and its effects in Europe in terms of collective anxiety, with regard to its relations with trust in scientific knowledge. This is an exemplary case with which to analyse the emotional state of anxiety generated by a sudden destabilizing event. Precariousness, uncertainty and insecurity have never been so evident – at least in recent times and in the Western world – as in the current pandemic condition (Lee, 2020). The Covid-19 pandemic crisis is an all-pervading source of anxiety, in social relations, in terms of impact on economic and healthcare systems, and for its capacity to reveal situations of scientific and practical ignorance. In this respect, the emotional spread of anxiety is related to the questioning of scientific knowledge, to the political capacity to react to the crisis rapidly, and to the necessity that individuals reorganize their everyday lives on the basis of unclear dispositions.

The pandemic crisis has transformed the anxiety related to potential and hypothetical risks into the concrete necessity to manage a present, ongoing, unknown event. No wonder that, to frame a simulacrum of control, the viral condition has been described with military language. The virus is the enemy; doctors and nurses the soldiers (celebrated or blamed); virologists and epidemiologists the generals that together with the government have planned the successful or failed strategies. This martial code has also been an attempt to cope with anxiety, encouraging the reactive capacities of all the actors involved. However, in the Western world, this communicative choice has often prevented a more dialogical and reflexive relation with scientific information, and the economic concerns often played against the scientific recommendations. The focus on scientific work in progress – rather than on its final results – and consequently on its more evident phases of uncertainty, discussion and errors, has fostered a state of anxiety related to disenchantment with scientific claims, sometimes also related to *infodemic*, the huge amount of information, true and false, circulating around the disease (Pulido et al., 2020).

## Modernity as the age of anxiety

In the cultural geography of emotions, anxiety has been considered as the most typical Western emotion. It is rooted in the history of European modernity, in its conceptualizations of subjectivity and autonomy, in its relationships with secularism and science, in its teleological and eschatological relationship with the future, and with its ambitions to control risks (Golob, 2017). Certainly, this does not mean that other cultures, other places and other historical experiences are not concerned by anxiety and distress, but there is an elective affinity with Western modernity and anxiety. This relationship has been at the centre of many philosophical investigations of modern culture since the nineteenth century, as well as of cultural, critical analyses from a non-Western point of view, such as those by the Indian sociologist and psychologist Ashis Nandy, who considers anxiety to be a typical modern and Eurocentric mood related to the loss of an imperial desire for control (Nandy, 1983, 2013).

In the Western cultural tradition, this affinity was first explicitly noted by Søren Kierkegaard in his essay *The Concept of Anxiety* (1844), a reference text for all the following philosophical reflections on this topic. While on the one hand Kierkegaard considered anxiety to be a feeling typical of the Christian – and more precisely Protestant – culture, on the other hand, he highlighted its mediating nature. Anxiety, he maintained, works as a process, as a medium between freedom and fear. This mediation is represented by the possibility, or the necessity, to make choices as the supreme privilege of the free modern individual. Thus, anxiety is related not only to uncertainty and to unforeseeable consequences, but also to the ability of a free person to decide in an autonomous way, independently of his/her community.

Just a few decades previously, in *The Phenomenology of Spirit* (1807), also Hegel had developed a fundamental insight into the relationship between the freedom of the modern individual and anxiety, based on his idea of negativity. For Hegel becoming a self-conscious person meant negating nature; the nature of the body, as well as of the environment, which becomes a mere resource for human activity. This negation is also a form of voluntary destruction, of dismemberment of a part of oneself, but for Hegel this was necessary to develop a life of the spirit able to overtake death. However, in everyday life this is often reduced to the effort to develop self-control, rationalization and predictability. Thus, this exposes the person to a self-negativity emotionally expressed by anxiety, an emotional disposition that Hegel called the ‘unhappy consciousness’.

Max Weber was the first to transform these philosophical reflections into a sociological analysis of anxiety as an emotional mood of the Western modern world. In his study on the Protestant ethic, he paid attention to the emotion of *angst* in the everyday life of the Pilgrim Fathers and of the first entrepreneurs on the American east coast (Weber, 2002 [1905]). Indeed, Weber does not use the notion of anxiety, but it is evident that angst becomes a general apprehension towards the future. Even though entrepreneurs’ attitude was related to religious belief – and characterized by feelings of incompleteness, guilt and awaiting – this had a special connection with their everyday life activities oriented to the future of investments, as signs of providence and predestination. The modern person shaped by Protestantism – as the cradle of capitalism – is inevitably an anxious individual making her/his choices alone. For Weber, this attitude was the precursor of the subsequent lay aspirations of the modern Western individual to control the future, and to gain mastery over nature through technosciences, as well as bureaucratic administration, legal formalism, and economic capitalism (Brubaker, 1992). Anxiety is not only a response to indeterminate dangers, but an emotion strictly related to angst as resulting from the consciousness of freedom (Valls, 2018). Since this historical turn, anxiety has proliferated on the ambitions of security and control in social and material life, and on the inevitable acknowledgement of the impossibility to control a growing variety of risks.

After the Weberian analysis, the connection between anxiety and Western modernity became even more explicit. It was discussed by the existentialists, under the influence of Heidegger and Sartre, as well as by the critics of modernity belonging to the Frankfurt School, and especially Adorno. Both approaches were oriented – in spite of their marked differences – towards a critique of the Enlightenment and its legacy of instrumental rationality. On the one hand, anxiety was framed as the basic emotion of a human condition trapped in the iron cage of reason and singularity; on the other hand, the Frankfurter critique of modernity considered anxiety to be the result of a society planned by economic structures, and as a malaise typical of a hyper-rationalized individual (Adorno, 1981 [1966]). The ‘vast programme’ of modernity was criticized both as a harbinger of performance anxiousness for the individual, and as a source of systemic paranoia related to its constitutive dualisms such as nature/culture, emotion/rationality, identity/otherness (Kosofsky-Sedgwick, 2003). It is not by chance that the counter-figure of modernity’s programme has been nihilism, that is, the contemplation of the non-sense of every choice, and the search for relief from anxiety in the abandoning of hubristic illusions (Giddens, 1991; Severino, 2016).

Reflection on the legacy of Enlightenment in terms of self-government and anxiety also characterized the last work of Michel Foucault – especially in his investigation of the ancient practice of *epimèleia heautoù*, as a method to counter anxiety as an emotional mood typical of the Western individual (Foucault, 2005) – even though an interest in anxiety was already present in his early work on *Mental Illness and Psychology* (Foucault, 1987 [1954]). In this analysis, Foucault focused on the idea of practice and techniques of the self as a guide towards an agency of the present, an ‘art of oneself’ drawn from Greek-Roman culture. Practices such as self-writing were popular at that time, as a way to prevent ‘a mental attitude turned toward the future which, due to its uncertainty, causes anxiety and agitation of the soul’ (Foucault, 1983: 8). Indeed, Foucault’s long digression on stoics and epicureans is also a reflection on anxiety; its pervasiveness could be considered as resulting from the ambiguities of self-government in neoliberal societies, where the injunction to conduct one’s conduct, to monitor one’s action, is produced through techniques where it is increasingly difficult to detect who is conducting whom. Alienated self-government is based on the anxious self-management of imminent risks, and on established social norms about what should be desirable. For Foucault, anxiety cannot be separated from the way in which human beings ‘are made subjects’, and later the challenging notion of parrhesia arose in his work as the main exercise of vigilance and resistance against anxiety and biopower.

Existentialism, Frankfurter critical theory and Foucault converge in describing anxiety as an emotion characteristic of Western modernity, but they also have in common a reflection on the relation of anxiety with immanence and contingency. ‘Practising the present’ is the acrobatic exercise of an individual obliged to cope with the unpredictability of the future, and the inconsistency of a teleological attitude of self-planning (Sloterdijk, 2013). To sum up, at the centre of the reflection on anxiety in modern Western culture there is the idea of agency as the possibility to make an autonomous choice and govern the situation, where the individual can decode the characteristics of the context, the stakes and values orienting the action, and being accountable for it. Anxiety is not only an emotion based on awaiting an uncertain future, or a response to stress; it is also the condition in which the modern Western person has been shaped, where individualization becomes a goal and an ongoing accomplishment, a form of discipline and the way in which individuals find self-fulfilment, self-gratification, and try to make sense of their experience.

## Anxiety in relation to uncertainty

While the history of Western modernity and its conception of the person have a specific relationship with anxiety, this emotional state seems today to have acquired a global cultural dimension. Many cultural features that originated in the West – from economic and financial structures to consumption habits – have become global characteristics with local adaptations in a global context of growing interdependences (Nederveen-Pieterse, 2012). A source of collective anxiety in a particular place, such as an economic default or an environmental disaster, can have an emotional and practical impact on a global scale. This is especially true when the source of anxiety is located, or has originated, in the ‘Global North’, but the acceleration of systemic emergencies (for example, terrorism, pandemic outbreaks, economic crisis, climate change) has blurred the geopolitical and geo-cultural distinction of anxiogenic sources (Moisi, 2009). Everywhere, the anxiety related to uncertainty is rationalized under the frame of the ‘exposure to risks’, and to the probability, distribution and characteristics of such risks.

This analysis was at the centre of the work of Ulrich Beck, who continued the German tradition of reflection on modernity and anxiety (Beck, 1992, 1998, 2009). According to Beck, the issue of risk management is the most evident connection between anxiety and the future in individualized societies, where the focus on risk is based on the removal of randomness, and on the endeavour to rationalize and plan. Beck defines risk as a systematic way to deal with hazards and insecurities, and anxiety as the dominant emotion in societies where risks correspond to waves of emergencies and threats, and to the impulse to govern imponderability. Risk is a modern notion related to calculation, evidence-based planning, and probability; it does not correspond to a generic threat. Risk means the anticipation of catastrophe. Even though the need to foresee the future is not only a modern requirement, and it is part of the human condition, modern societies and their scientific method based on statistics and stochastic damage to foresee possible negative events have a much more intense relationship with the ideas of security, safety and certainty.

Indeed, Beck’s notion of the risk society has spread as a theoretical interpretation with a systemic extent, even though it is culturally valid mainly for Western societies, and this analysis takes the German cultural response to global risk as a model to write about the risk society as a whole. As Beck acknowledges (Beck, 2005), an approach in terms of risk society does not have the same effect and the same features in different cultural contexts, and many countries cannot afford to be a ‘risk society’. Moreover, there are different cultural attitudes towards risk and consequently different emotional reactions, not to mention that there are differences in terms of individual attitude, education, social environment, gender or age (Lupton, 2013). Yet, according to Beck, globalization includes different cultures of risk perception not only because of objectively different exposures to risks; in Western culture risks exist in a permanent state of ‘virtuality’. Consequently, in the Western world anxiety is related to a constant visualization and anticipation of risks independently of their probability.

This relation between the emotional state of anxiety and an approach in terms of systemic risks is presented by Beck as both a cultural and an ontological claim; risk is at the same time a grid of interpretation and an ontological status. On the one hand, today risks are increasingly systemic and potentially global (nuclear accidents, virus pandemics, climate change); from a material point of view, there are no longer safe places immune from at least the indirect consequences of risks of this kind. On the other hand, risk is a cultural frame of analysis; regardless of the kind of risks, the Western cultural attitude is that of identifying, controlling and reducing them.

Even though Beck’s approach has been criticized for its excessive emphasis on the relation between anxiety and risk (Rabsborg, 2012), what is characteristic of this relation in Western culture is its basis in individualization as self-management, as it was already intercepted by the critique of modernity developed by Sartre, Adorno and Foucault. Hence, the sensitivity to risk, and the anxiety correlated with it, cannot be understood without considering the cultural injunction to self-government: that is, a psychological attitude of self-accountability whereby the single individual is required to find a solution, or to take a decision, also in regard to problems outside his/her range of control or comprehension. Public agencies – education systems, employment services, welfare state – private enterprises, political and media discourses, experts, relatives, friends and guidebooks encourage the individual to seek autonomy and self-realization so that s/he is ready to adapt to unexpected changes. The singularized individual is surcharged of new tasks of self-protection and self-management with an inevitable emotional consequence in terms of anxiety (Martuccelli, 2010). Consequently, anxiety is related not only to the requirement to control risks but also to a widespread process of singularization and to an absence of representations of the future.

Besides individualization and virtual anticipation of risks, there are other typical Western ways to frame anxiety. The role of technoscience, techno-bureaucracy and scientific expert knowledge was widely anticipated by Weber, Heidegger and Gelen: modernity produces technological risks and the necessity to control them with other technological and institutional tools (Sloterdijk, 2013). We find a similar analysis in the work of Anthony Giddens, who explicitly considers anxiety as the emotional state typical of Western modernity, but who also describes a bond between anxiety and trust in the numerous varieties of expert knowledge characterizing the modern condition. Trust and anxiety are future-oriented emotions based on confidence in one’s ability to evaluate the situation. Hence, they are also related to the circularity of reflexivity (Giddens, 1990, 1991).

Anxiety and trust are related at a personal level as well; according to Giddens, the lack of trust and security in everyday life and personal relations may generate a pervasive feeling of anxiety. More than to risk, anxiety is related to the feeling of security and trust in the quality of control; that is, to the degree of trust in all the countless processes beyond our control, not only technological but also personal. Whereas to look at new technologies without anxiety we can perhaps trust expert knowledge, to trust personal relationships we can no longer rely on clear social rules and pathways. In Giddens’ analysis there is no clear-cut distinction between social relationships of trust, and trust in expert systems, because they are both related to the construction of a ‘public trust’. Indeed, anxiety and trust share the ‘absence of certainty’: trust implies the absence of information about the other reliability as emotional cost (Barbalet, 2009).

As we shall see in the following sections, this is especially evident in relation to scientific knowledge. Science cannot present itself as neutral and value-free, and trust in scientific expertise – especially in the biomedical field – is always based on the asymmetry of knowledge between the expert and the ‘layperson’, and on the related trust of the latter based on his/her incapacity to verify such expertise technically, which again entails the necessity to trust an expert system (Giddens, 1990). Trust – at least in modern Western culture – always operates in an environment of contingency and insecurity, where confidence in experts and in everyday routines may be disappointed. This frustration of trust can range from simple scepticism to existential angst and unbearable anxiety.

Giddens describes anxiety as a general social state engendered by an increasing lack of trust in both the project of modernity and in expert knowledge (Giddens, 1990). Yet, trust in science and scientific knowledge has a specific relation with anxiety as an emotional state of modernity, especially in regard to the biomedical field and research on diseases. The recent Covid-19 outbreak can be considered a test-bench on which to analyse the historical legacy of the relation of Western modernity with anxiety, and to explore the connections of anxiety with acknowledgement of the limits of scientific knowledge and governmental control.

More than an alleged threat, or hypothetical risk, the pandemic crisis has been a sudden and systemic experience of loss of control, as well as a generator of new forms of governmentality. In order to shed light on the characteristics of anxiety, the systemic effects of the Covid-19 outbreak can offer different perspectives: economic instability, unemployment, crisis of the hospitals’ organization, protest and social unrest, to name but a few. In the following two sections we focus on a more specific perspective: the analysis of the relations between anxiety and the trust in scientific knowledge, and its capability – or incapability – to manage the viral condition.

## Anxiety and ignorance: The Covid-19 outbreak in Europe

The approach to anxiety based on Beck’s notion of the risk society usually refers to generic global threats, such as climate change, nuclear catastrophes or international terrorism. Periodic international surveys, such as Eurobarometer, can register the variations of European public opinion regarding the main sources of anxiety and their geography (Eurobarometer, 2019). Before the Covid-19 outbreak, unemployment was the main source of anxiety in Europe; immigration followed in the list of worries; international terrorism was a matter of concern in the European countries hardest hit by terrorist attacks like France and the UK, but also ones not involved like Italy; while climate change was one of the main sources of anxiety among young people.

Yet, in Europe, climate change, a nuclear disaster or even a terrorist attack were considered mainly as virtual risks, long-term threats, or as events unlikely to occur. Such matters of concern were associated with a degree of anxiety similar to background noise, an emotional malaise that in some cases – such as that of environmental crisis – could be exorcized with everyday good habits such as an organic diet, ethical consumerism, bike sharing or electric cars. According to a recent Gallup survey (Kluch, 2020), the outbreak of a contagious disease was already present among the European worries long before the Covid-19 pandemic began. Europeans remembered previous pandemic crises, such as ‘bird’ or ‘swine’ flu, but this was only a secondary matter of concern, following immigration, unemployment or terrorism.

The Covid-19 emergency has been described as the greatest mass emotional event since the Second World War, generating a global epidemy of anxiety. In the media, psychologists have been the second most consulted scientific category after physicians and virologists, and the social media have been invaded by narratives of individual crises of anxiety, or recommendations on how to deal with it during the lockdowns. Indeed, the pandemic crisis has generated anxiety in diverse ways: anxiety about the health risk, of course, but also in relation to social and familiar distance, worries about unemployment and losing one’s job, anxiety generated by the incapacity of scientists to give instant answers to a public accustomed to rapid responses, or anxiety provoked by the frenetic circulation of fake news, conspiracy theories, fanciful and miraculous treatments. More than to an imperative of control, as in Beck’s analysis, this anxiogenic wave was mainly related to people’s sudden discovery of the extent of their ignorance and unpreparedness to deal with the viral condition. The global spread of the disease has generated an anxiety no longer related to the attempt to foresee and prevent fatal risks but to acknowledgement of an inability to understand processes that they considered taken for granted.

Therefore, the public image and the trust in the role of scientific and biomedical knowledge during the Covid-19 outbreak are factors essential for understanding the role of anxiety within the modern legacy. Actually, in the modern paradigm, we are supposed to live in a knowledge-based society (Beck, 2009), but there is always an anxiety-inducing moment – like that of the 2020 pandemic crisis – when we discover that we live in a ‘ignorance-based society’. Ignorance is related to uncertainty and to risk, to the management of the ‘unknown’, but at the same time it is itself an implicit product of modern scientific knowledge, whose characteristic is that it is always in progress. For science, more than a dark space of inaction, ignorance is a point of departure. Nonetheless, European citizens expected an immediate reaction by scientific knowledge and a rapid response by the health system to the Covid-19 crisis; and they soon realized that scientific research needs time, and it works by trial and error, while health systems are bureaucratic machines with many organizational holes. Anxiety generated by the virus outbreak seems to be related to the sudden shock of societies accustomed to considering the risk of zoonosis as they consider environmental catastrophes: that is, background hypothetical risks that can be addressed by means of technoscience and anthropotechnics. The modern pragmatic approach in terms of technoscience problem-solving – we produce problems but we are able to find solutions to them – and in terms of self-governmentality, has been destabilized by a systemic emergency involving all the structures of social life, and impossible to frame within a single perspective.

The relation between modernity and anxiety in terms of government and control of risks is faced with a destabilizing event – such as Covid-19 – able to produce entropic connections of risks, without separation of causes and effects (Latour and Weibel, 2020). Anxiety appears as the emotional state of bewilderment caused by the impossibility to reconstruct the complex chain of such connections; this is especially evident in relation to the trust in biomedical knowledge, where anxiety – in the form of disorientation, suspicion and frustration – has often replaced confidence in the scientific expertise as beacon of modernity. The anxiety generated by the impact of Covid-19 on the public image of biomedical knowledge – in terms of effectiveness and responsiveness – is an unprecedented historical case.

## Anxiety and trust in scientific knowledge

Scientists have never been so exposed to the media as they have been in the Covid-19 outbreak. Requested but also criticized, considered as the generals in the war with the invisible enemy – immediately described as an ‘otherness’ – they have been obliged to open their laboratories to the spotlight of the media, and to issue periodic reports on their work in progress. Yet, what is taken for granted in science – uncertainty is a resource and results can be refuted – is a source of anxiety for the politics of everyday life. Scared by the virus, citizens need rapid solutions, because modern culture has taught them that scientific work is a ‘function’ of the social system and it is there to solve problems (Mol, 2002). While there is a vast literature in STS studies on the relationship between science, politics and public opinion, the emotions related to the perception of biomedical knowledge and the expectations in its regard have been less explored, even though they are crucial for a sociology of anxiety in modern Western societies.

According to the most recent Gallup Global Monitor (2019), a global-scale survey of public attitudes to science and health, there are different degrees of knowledge and perception of scientific and biomedical functions around the world. Nevertheless, and regardless of the biases of gender, age, education, income and country of residence, around 90% of all the interviewees agreed that to study disease is a fundamental part of scientific work; people expect science to give answers to health problems. In Europe the trust in this capacity and related benefits was very high (around 85%), as well as the trust in doctors and medical staff (90%).

Overall, during the Covid-19 outbreak, this trust has not been undermined by the difficulties of many national health systems to cope with the pandemic crisis. Still, in Europe trust in biomedical expert knowledge is progressively diminishing, and this is mainly related to the patterns of public communication and participation, and to the sources of knowledge. For example, in recent decades, the financial involvement of private scientific companies at the expense of public social concerns has been among the first sources of scientific distrust, while social networks have had a great impact on the construction of trust and distrust in expert scientific knowledge, since the Web is the primary source of information about science and technology (Huber et al., 2019; Jasanoff, 2015). The mass media are especially influential in the field of the danger and treatment of infectious diseases, even more when the disease is new and unknown, so that the danger it might provoke is difficult to frame and to measure. Consequently, this fosters an anxiety generated by the urgency of finding alternative sources of information, reassurance and practical remedies (Broomell and Kane, 2017).

The difficulties of scientists in communicating with different publics has been an additional obstacle to constructing trust in their work (Barbalet, 2011; Drummond and Fishoff, 2020). This is especially evident in the case of emerging infectious diseases, as already happened for AIDS (Joffe, 2011), and now with Covid-19. These are pandemic infections producing high degrees of anxiety but also of ‘emotional fatigue’, because of evident social effects such as economic impact, physical distancing, social blaming of particular behaviours for the disease’s origin and spread, as well as a possible stigmatization of those who have contracted the disease or who are believed to have intensified its spread.

What happened with the outbreak of the Covid-19 emergency was a convergence of all the difficulties in risk evaluation, with the lack of information about the disease and the impossibility to give coherent information to the public. To weigh benefits and risks about cure and prevention, to balance health protection and economic interests, was difficult because there was so little knowledge about the virus, and the public was obliged to follow day-by-day the contradictory and awkward experimental ‘kitchen of science’ (Latour and Weibel, 2020).

Hence, the relation between anxiety and trust in scientific work during the pandemic crisis has been deeply involved in communicative misunderstandings. In relation to the Covid-19 emergency, every person has had her/his own personal perspective, based on exposure to risk, experience of the disease, or exposure of the local environment to the virus. The emotional interpretation of scientific work has been filtered by this personal experience, and by the perception of and trust/mistrust in institutional work – for example concerning lockdown policies. Anxiety has been the psychological cost of trust in a scientific knowledge, and in institutional decisions full of contradictions and systemic vulnerabilities.

While modern culture considers scientific work as an efficient problem-solving function of the social system, since the 1980s a sequence of new viral diseases, such as AIDS, Ebola, or the succession of ‘bird flu’ and ‘swine flu’ – changed this social perception of invincibility, at least for biomedical knowledge. An important novelty of these infectious diseases was their pandemic nature, their rapid spread in a globalized world, and their great media visibility due to their virality. Compared to the anxiety associated with other health risks, such as cancer or stroke, referring to fatality or lifestyles, the public awareness of and engagement with the new infectious diseases fluctuates and is related to specific alarms and media framing of the risk, with an emotional pattern of peaks and troughs of attention (Joffe, 2011; Smith, 2006). For European public opinion, Covid-19 was not totally a surprise, even though the extent and rapidity of its spread were unexpected. However, the exceptionally widespread media coverage of the viral outbreak fostered a new expression of anxiety, reflexively oriented towards constant examination of practices in the light of incoming, and often contradictory, information.

## Conclusion

As a sort of intimate enemy, anxiety has been considered the inconspicuous but pervasive emotional state of Western modernity, with its ambitions of governmentality, control, planning, and its doubtful trust in expert systems. The critique of modernity developed in Western philosophical thought of the twentieth century was often intertwined with the analysis of an emotional state of anxiety about the failures, contradictions or excessive ambitions of the modern project. Since then, the ironic nihilism of postmodern discourse or the analysis of the complexity, uncertainty and risks related to globalization processes have also explicitly addressed the question of anxiety. Whilst at the beginning of modern society anxiety was mainly a private feeling of subjective malaise, in postmodern and post-industrial societies, it is a collective emotion based on the awareness of the potential catastrophes that we are generating.

The state of exception provoked by the Covid-19 outbreak is probably going to introduce a new turning point in this reflection on Western culture and its emotional anxiety-inducing accompaniment. This is evident not only with respect to the conditions of health and economic systemic risks induced by the virus, but also with respect to the intertwining among scientific knowledge, political government and economic decisions. The classic modern separation of systemic functions like science, politics and economics – each with its own internal logic – or the binarism between nature and politics, is no longer plausible.

While, on the one hand, the ontological security related to scientific and biomedical knowledge has been compromised by the acknowledgement of unanticipated risks out of control, on the other hand, an overall decreasing trust in the capacity to find technical and governmental solutions to crises can be associated to *infodemic* and fake news, as well as by the close intertwining of social inequalities and systemic risks. The Covid-19 outbreak presented all the typical characteristics of a global risk: it was not limited to one geographical location; its consequences were incalculable and at least partially irreversible; it was predicted but it was impossible to prevent it; it was a source of collective anxiety because of its elusive nature, difficult to frame and to control, or to be domesticated by science. Such unveiling of ignorance is in contrast with the efforts of control, which were consequently expanded and technologically sophisticated.

Hence, anxiety arises from the controversies related to political and technical decisions taken not only in a situation of absolute uncertainty and unpredictability, but also amid the impossibility of an internal control based on the community or the nation-state. The legal and normative space of the nation-state can take decisions about the emergency but not about the control of the event itself, whose nature is global and boundless. The pandemic crisis has been a paradoxical and involuntary medium of transnational communication, a material contagion difficult to contain, demonstrating the cultural fiction of borders and communities.

For all these reasons, the emotional consequences of Covid-19 will probably have an impact on the modern Western legacy and its relationship with anxiety, reinforcing the ambivalent relations with expert knowledge and technoscience, the obsessive anticipations of risk, the necessity of individualized responses to uncertainty, the disenchantment and inconstancy in regard to assertions of truth, and the focus on immanence and contingency rather than on planning futures. With a global reverberation, the ‘scars of the spirit’ left by the pandemic crisis confirm the centrality of anxiety as the main emotional legacy of Western modernity.
